# HIV-1 tat and rev upregulates osteoclast bone resorption

**DOI:** 10.7448/IAS.17.4.19724

**Published:** 2014-11-02

**Authors:** Nicholas Chew, Eemin Tan, Lei Li, Ryan Lim

**Affiliations:** 1 Infectious Diseases, National University of Singapore, Singapore; 2 Obstetrics and Gynaecology, National University of Singapore, Singapore

## Abstract

**Introduction:**

Disruption in bone homeostasis with increased osteoclastic resorption may lead to osteoporosis. HIV tat has been found to increase differentiation of precursor cells into osteoclast (OC) [1]. Presence of soluble HIV proteins in virally suppressed HIV patients on ART may drive a bone resorption phenotype. We investigated the role of soluble HIV proteins (tat, gp120 Mn and Bal, rev and p55-gag) on osteoclastogenesis and OC resorptive capacity.

**Methods:**

Mouse monocyte RAW 264.7 cells were cultured in vitro and induced to differentiate into OCs with 50 ng/mL RANKL and 25 ng/mL mCSF. Medium was supplemented with 100 ng/mL of recombinant HIV tat, gp120 (Mn and Bal), rev, nef and p55-gag, respectively, with zolendronate as negative control. Differentiated OCs were stained for TRAP and counted. OC resorption function was examined by culturing differentiated OCs (in the presence of respective HIV proteins) on dentin-coated plates and examining the following (i) sealing zone formation, (ii) volume of resorption pits and (iii) area of resorption pits per field using confocal microscopy. Expression of OC specific genes including NFATc1 and cathepsin K was investigated by qPCR. Reactive oxygen species (ROS) production is essential in RANKL-induced OC differentiation [2,3]; effect of these proteins on ROS production was assessed using the fluorescent H2DCFH-DA. Mean fluorescence intensity was then measured by flow cytometry. TNFα production by OC precursors when incubated with tat and rev was measured by ELISA.

**Results:**

Tat and rev treatment was associated with increased OC formation by 70 and 26%, respectively (p<0.01), relative to control, while zolendronate significantly inhibited OC formation by 75%. Gp120 Mn and Bal, nef and p55-gag treatment had no effect on OC differentiation. Interestingly, neither tat nor rev treatment caused significant increases in sealing zone formation but increased dentin resorption pit area by 28 and 19%, respectively, and resorption pit volume by 11 and 6%, respectively. Tat protein treatment was associated with upregulation of NFATc1 and cathepsin K mRNA expression by 20 and 15%, respectively. Incubation with tat and rev led to a dose-dependent increase in intracellular ROS production in the monocytes and OC precursors and significant upregulation in TNFα cytokine production by the OC precursors.

**Conclusions:**

In addition to their effect of OC differentiation, we demonstrated the effects of tat and rev on OC resorption. HIV tat and rev are both biologically active in driving a pro-osteoclastic phenotype.
